# Alanine Substitution Inactivates Cross-Reacting Epitopes in Dengue Virus Recombinant Envelope Proteins

**DOI:** 10.3390/v12020208

**Published:** 2020-02-13

**Authors:** Viviana C. Zomosa-Signoret, Karina R. Morales-González, Ana E. Estrada-Rodríguez, Ana M. Rivas-Estilla, M. Cristina Devèze-García, Edgar Galaviz-Aguilar, Román Vidaltamayo

**Affiliations:** 1 Departamento de Bioquímica y Medicina Molecular, Facultad de Medicina, Universidad Autónoma de Nuevo León, Monterrey NL 64460, Mexico; 2 Departament of Basic Science, School of Medicine, Universidad de Monterrey, Av. Ignacio Morones Prieto 4500 Pte, Garza García NL 66238, Mexico

**Keywords:** epitope inactivation, dengue, Zika, serological methods

## Abstract

The expansion of the habitat of mosquitoes belonging to the *Aedes* genus puts nearly half of the world’s population at risk of contracting dengue fever, and a significant fraction will develop its serious hemorrhagic complication, which can be fatal if not diagnosed properly and treated in a timely fashion. Although several diagnostic methods have been approved for dengue diagnostics, their applicability is limited in rural areas of developing countries by sample preparation costs and methodological requirements, as well as cross-reactivity among the different serotypes of the Dengue virus and other flavivirus, such as the Zika virus. For these reasons, it is necessary to generate more specific antigens to improve serological methods that could be cheaper and used in field operations. Here, we describe a strategy for the inactivation of cross-reacting epitopes on the surface of the Dengue virus envelope protein through the synthetic generation of recombinant peptide sequences, where key amino acid residues from Dengue virus serotype 1 (DENV-1) and 2 (DENV-2) are substituted by alanine residues. The proteins thus generated are recognized by 88% of sera from Dengue NS1+ patients and show improved serotype specificity because they do not react with the antibodies present in seroconverted, PCR-serotyped DEN-4 infected patients.

## 1. Introduction

Dengue and other mosquito-borne viral diseases threaten heavily populated areas around the world, and the expansion of the habitat of their vector and an increase in human mobility lead to the introduction of novel infectious agents, which pose new challenges to national health services, as the rapid spread of Chikungunya and Zika viruses in the Americas has demonstrated [1,2].

Four serotypes of Dengue viruses (DENV1 through DENV4) have been identified. In naturally occurring infections, acquired immunity against one serotype rarely protects against a subsequent secondary infection by any of the other serotypes [3,4,5], but the presence of circulating, non-neutralizing antibodies against any of the serotypes increases the risk of severe hemorrhagic fever/shock syndrome in patients infected by a different serotype in a secondary or concurrent exposure [4,5,6]. 

The envelope E protein of the DENV virion is a major antigen determinant of its serotypes and serves as a target for most of the antibodies generated by patients in naturally occurring infections [5,7]. The E protein of the Flaviviridae family, to which DENV belongs, is organized into three rigid, beta-sheet-rich structural domains (I-III) that are linked by semiflexible loops [8,9,10,11]. 

E proteins form dimers that, along with the membrane M proteins, cover the entire surface of the virion. The E protein mediates receptor binding and fusion during infection. Environmental acidification upon cell internalization into the endocytic pathway leads to a conformational change of the E proteins that trimerize at low pH, which allows for the insertion of the E protein into the membrane of the endolysosomes and the escape of the genetic RNA material of the virion into the cytosol [8,9,11,12]. 

These conformational changes are necessary for infectivity and are mediated by a loop at the tip of Domain II, which is well preserved in all members of the Flaviviridae family and is recognized by cross-reacting antibodies present in sera from patients infected by various flaviviruses [13]. 

Epitopes that also elicit cross-reacting antibodies against all four serotypes of DENV have been identified in all three structural domains of the E protein, interspersed with other serotype-specific epitopes [14,15,16,17]. The presence of these cross-reacting epitopes prevents the use of recombinant full-length E proteins in serological diagnostic tests for DENV. Derivatives of non-structural Protein 1 (NS1) are currently used in serological tests approved by health systems worldwide [18,19,20,21]. However, the expansion of the Zika virus (ZIKV) out of its original zones of high prevalence has shown the cross-reacting properties of Dengue NS1 tests against this related flavivirus [22].

The co-circulation of flaviviruses (DENV, ZIKV) and other arboviruses (Chikungunya virus) that share common mosquito vectors and cause febrile syndromes with common symptoms that impair clinical criteria-based diagnosis, underscores the demand for better screening tests that allow for rapid identification of etiological agents. Here, we describe a strategy for the inactivation of cross-reacting epitopes in recombinant derivatives of DENV E protein through the alanine substitution of key amino acid residues in synthetic E protein sequences.

## 2. Materials and Methods 

### 2.1. Virtual Mutagenesis through Molecular Modeling Based on Crystal Structures of DENV-1 and 2 E Proteins

Crystal structures for DENV-1 (ID: 4GT0) [11] and DENV-2 (ID: 4UTC) [23] E proteins were obtained from the Research Collaboratory for Structural Bioinformatics Protein Data Bank (RCSB PDB) and visualized on the Swiss PDB viewer software suite [24]. Epitopes for which antigenic determinants were identified at amino acid residue level [15,16,17,25] were labeled on the model and their solvent-accessible profile was evaluated using the surface generating tool. Acidic (D, E), basic (K, R), aromatic (F, W, Y) or proline (P) residues in cross-reacting epitopes [26] were substituted in silico by alanine (A) residues using the mutation tool. Energy minimization of the resulting protein sequence and structure prediction fitted to the available crystal was performed using the Swiss model software and server [27,28]. Renders superposing the mutant over the wild-type structures were created using PyMOL (OS X v. 2.0.5 Schrödinger; San Diego, CA, USA).

### 2.2. Generation of Synthetic Recombinant DENV E Proteins

The amino acid sequences that disrupted cross-reactive epitopes, but not the overall structure of the E protein monomers, were selected for expression. A synthetic DNA sequence coding for the mutant proteins was designed by the introduction of an appropriate translation start sites and stop codons, and coding codons were optimized for expression in *Escherichia. coli*. The induction of the expression of full-length mutant E proteins leads to cytotoxic effects that were overcome by the elimination of the last 85 amino acids at the carboxy-termini of the mutant E polypeptides. The resulting final sequences were expressed in *E. coli* and purified by affinity chromatography using a 10x His-tag introduced at the N-terminus of each protein. Synthetic DNA sequences and mutant recombinant E proteins with preserved serotype-specific epitopes corresponding to DENV-1 (synthDENVElope 1) and DENV-2 (synthDENVElope 2) were produced by Genscript (Piscataway, NJ, USA). Control recombinant E proteins from DENV-1, generated in insect cells (cat. No. DEN-033), or DENV-2, generated in *E. coli* (cat. No.DEN-022), were purchased from Prospec (East Brunswick, NJ, USA).

### 2.3. Sequence of the Synthetic DENV E Proteins

#### 2.3.1. syDENV-1 Product

MRCVGIGNRDFVEGLSGATWVDVVLEHGSCVTTMAKDKPTLDIELLKTEVTNPAVLRKLCIEAKISNTTTDSRCPTQGEATLVEEQDTNFVCRRTFVDRGWGNGCGLFGKGSLITCAKFKCVTKLEGKIVQYENLKYSVIVTVHTGDQHQVGNETTEHGTTATITPQAPTSEIQLTDYGALTLDCSPRTGLDFNEMVLLTMKKKSWLVHKQWFLDLPLPWTSGASTSQETWNRQDLLVTFKTAHAKKQEVVVLGSQEGAMHTALTGATEIQTSGTTTIFAGHLKCRLKMDKLILKGMSYVMCTGSFKLEKEVAETQHGTVLVQVKYEGTDAPCKIPFSSQDEKGVTQNGRLITANPIVTDKEKPVNIEAEPPFGESYIVVGAGEKALKLSWFKKGSSIGKMFEATARGARRMAILGDTAWDFGSIGGVFTSVGKLIHQIFGTAYGVLFSGVSWTMKIGIGILLTWLGLNSRSTSLSMTCIAVGMVTLYLGVMVQA

#### 2.3.2. syDENV-2 Product

MRCIGMSNRDFVEGVSGGSWVDIVLEHGSCVTTMAKNKPTLDFELIKTEAKQPATLRKYCIEAKLTNTTTESRCPTQGEPSLNEEQDKRFVCKHSMVDRGWGNGCGLFGKGGIVTCAMFRCKKNMEGKVVQPENLEYTIVITPHSGEEHAVGNDTGKHGKEIKITPQSSITEAELTGYGTVTMECSPRTGLDFNEMVLLQMENKAWLVHRQWFLDLPLPWLPGADTQGSNWIQKETLVTFKNPHAKKQDVVVLGSQEGAMHTALTGATEIQMSSGNLLFTGHLKCRLRMDKLQLKGMSYSMCTGKFKVVKEIAETQHGTIVIRVQYEGDGSPCKIPFEIMDLEKRHVLGRLITVNPIVTEKDSPVNIEAEPPFGDSYIIIGVEPGQLKLNWFKKGSSIGQMFETTMRGAKRMAILGDTAWDFGSLGGVFTSIGKALHQVFGAIYGAAFSGVSWTMKILIGVIITWIGMNSRSTSLSVTLVLVGIVTLYLGVMVQA

### 2.4. Sera Sample Collection

The experimental protocol was approved by the Research and Ethics Committee at Universidad de Monterrey (Protocol 092012-CIE, 22 February 2012). Sera samples from patients identified by the Department of diagnostics of the Laboratorio Estatal de Salud Pública de Nuevo León were collected from February to December 2015. Samples were collected after informed consent was obtained from each patient and all samples were coded for anonymity. The presence of the NS1 protein was determined using the Platelia Dengue NS1 kit (cat. 72830, BioRad, Carlsbad, CA, USA). The presence of anti-NS1 IgM and IgG antibodies was determined using Panbio capture ELISA kits (cat. nos. E-DEN01M and E-DEN02G. Alere North America, Orlando, FL, USA), following the Mexican national standards for Dengue diagnostics stated in Norma Oficial Mexicana NOM-032-SSA2-2010, para la Vigilancia Epidemiológica, Prevención y Control de Enfermedades Transmitidas por Vector (https://www.gob.mx/salud/documentos/norma-oficial-mexicana-nom-032-ssa2-2010). Naïve sera were obtained from patients presenting to the service with febrile symptoms but were negative for all three previously described Dengue NS1 (antigen plus IgM and IgG) tests. Samples from a total of 100 naïve sera and from 130 anti-NS1 IgG positive sera were identified, collected, aliquoted and stored at −70 °C until use. The pooled chemiluminescence signals of these presumptive naïve sera were used as the baseline reference of the ELISA tests and are depicted by the dark blue boxes appearing in Figures 4 and 5. 

PCR serotyping of appropriate samples was performed following CDC recommendations [29]. DENV-4 positive serum samples were obtained from Laboratorio Estatal de Salud Pública de Yucatán in Southern Mexico. The seroconversion of PCR-positive patients was determined with the anti-NS1 Panbio IgG capture ELISA kits described above. February.

### 2.5. Chemoluminescence-Dependent Enzyme-Linked Immunosorbent Assay

**Figure 1:**
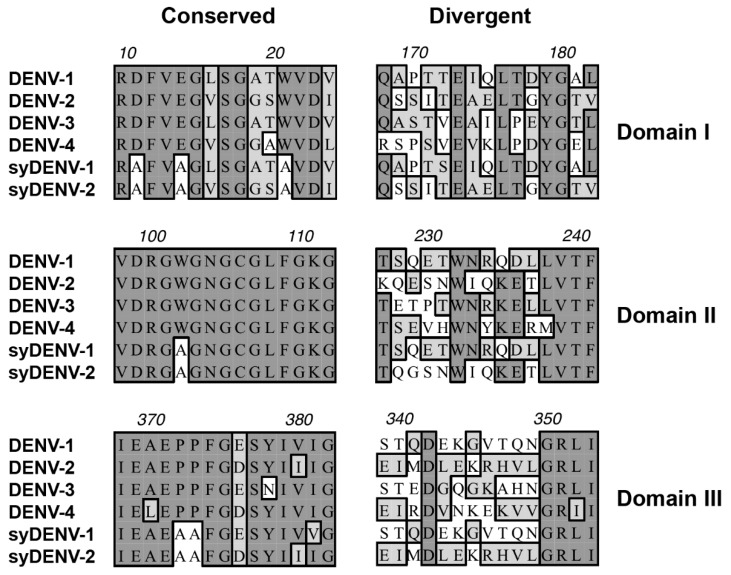
Linear cross-reacting and serotype-specific epitopes within the sequence of the DENV E protein. Examples of cross-reacting epitopes located across the three domains of the DENV E protein show highly homologous amino acid sequences shared by all four serotypes of DENV (Conserved column on the left). Proline, aromatic, and/or charged amino acid residues within these cross-reacting epitopes were substituted for alanine in synthetic DNA sequences of the mutant E proteins (the empty boxes around As in syDENV protein sequences). The sequences of serotype-specific epitopes (Divergent column on the right) remained unmodified in the synthetic protein sequences.

Synthetic recombinant E proteins were resuspended in sodium carbonate buffer (100mM, pH=9.6) at a final concentration of 50 ng/µL and were incubated (100 µL/well) overnight at 4 °C in 96-well polystyrene ELISA plates (cat. no. 3361, Corning Inc., Corning, NJ, USA). Wells were then washed 3 times for 5 min (3 × 5′) in phosphate-buffered saline (PBS; 10mM phosphate buffer, 150 mM NaCl, pH=7.4) supplemented with 0.05% Tween 20 detergent (PBS-Tween). Next, the wells were blocked for 30 min with 100 µL of bovine serum albumin solution (1% in PBS-Tween). After washing the wells (3 × 5′ with PBS-Tween), 100 µL of sera sample diluted 1:500 in PBS-Tween was added per well, in triplicate, and plates were incubated overnight at 4 °C. In a series of experiments to determine the antigenic activity of the recombinant mutant proteins, patient sera were substituted by a murine monoclonal antibody vs. DENV-2 (clone NYRDeng2. Cat. no. ANT-143; Prospec) diluted 1:1000 in PBS-Tween. The baseline of these tests was determined by the reaction of a murine monoclonal antibody in wells containing no recombinant protein, as shown in Figure 1. 

Plates were then washed 3 × 5′ with PBS-Tween, and 100 µL of horseradish peroxidase-labeled secondary antibody diluted 1:25,000 in PBS-Tween was added per well. Secondary antibodies (donkey anti-mouse IgG; cat. no. 715-035-150 and donkey anti-human IgG; cat. no. 709-035-149) were purchased from Jackson ImmunoResearch (West Grove, PA, USA). After a 30 min incubation at 25 °C, plates were washed 3 × 5′ with PBS-Tween and once for 5 min with PBS. Finally, 100 µL of SuperSignal ELISA Femto luminol substrate (cat. no. 37075; Thermo Fischer, Grand Island, NY, USA) was added per well, incubated for 1 min, and plates were read under a Glomax multi-detector plate reader (Promega; Madison, WI, USA) using the luminescence module with an integration of 0.5 s per well.

### 2.6. Statistical Analysis

Data distributions are represented as box plots. The median of the population is depicted by a solid line, while the mean is depicted by a dashed line, within the boxes. The lower border of the boxes represents the 10%, while the upper border represents the 90% percentiles of the data distribution. Whiskers extend towards the 5% and 95% percentiles of the data distribution. 

Group medians were compared using the non-parametrical Kruskall–Wallis ANOVA on ranks test, followed by multiple comparison posthoc tests. Student-Newman-Keuls (SNK) for groups of equal size and Dunn for unequal-size groups using the Sigmastat tools from Sigmaplot (v. 10.0; Systat Software, San Jose, CA, USA). 

## 3. Results

### 3.1. Alanine Substitutions Clustered in Two Separate Motifs on Domains I and III and on the Fusion Loop of Domain II of the E Protein

The amino acid sequence of E protein is highly preserved among all DENV serotypes, and, for that reason, several linear cross-reacting epitopes can be identified along the three domains of the E protein. Some of these cross-reacting determinants are also preserved among other flaviviruses, such as those conforming the fusion loop at the tip of Domain II (Figure 1 middle panel, amino acids surrounding W101 in Domain II) [9,11,30,31,32]. By in silico modeling of the structure of the E proteins from DENV-1 and DENV-2, we identified the cross-reacting epitopes on the surface of the E proteins and then systematically substituted either proline, charged or aromatic amino acid residues present in the cross-reacting linear epitopes for alanine in order to alter their structure and inactivate them [17,26,33]. Those substitutions that lead to the alteration of the solvent accessible structure of the cross-reacting epitopes, with minor perturbations to the overall 3D structure of the protein model, were selected for the generation of mutant versions of the E proteins through synthetic DNA sequence generation and expression in an *E. coli* heterologous system. 

The interspersed serotype-specific epitopes were spared (Figure 1, right panel) to increase their contribution in serological tests. Two synthetic variants of these mutant E proteins were generated: one was based on the amino acid sequence of the DENV-1 E protein (syDENV-1) and the other one was based on the sequence of the DENV-2 E protein (syDENV-2), each preserving the serotype-specific epitopes of its corresponding parental sequence.

**Figure 2:**
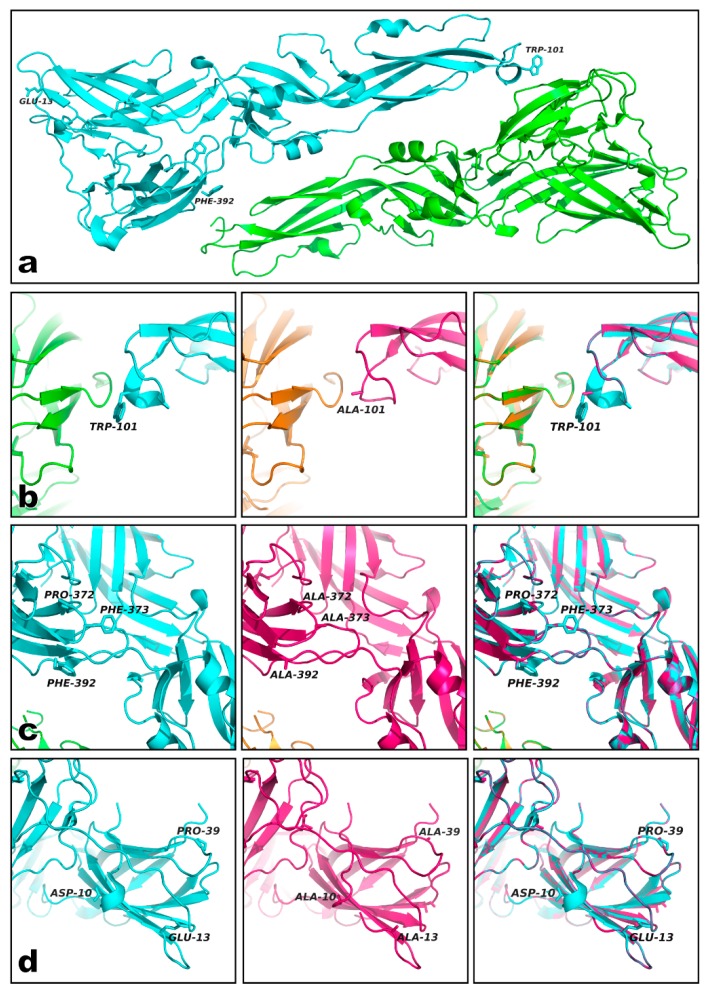
Alanine-substituted epitopes clustered in three regions of the DENV E protein. Alanine-substituted epitopes clustered in three separate regions at the tips of Domain I (Glu13 in **a**), II (fusion loop around Trp-101 in **a**), and III (Phe-392 in **a**). Examples of the alteration of solvent-accessible surfaces induced by the alanine substitutions are shown in panels (**b**–**d**). The introduction of alanine residues modified the solvent-accessible surface of the cross-reacting epitopes by either changing the orientation of the amino-acid side chain (cyan Trp-101 vs. pink Ala-101 in **b**) or the plane of rotation of the ß-sheets constituting the domains (cyan vs. pink structures in **c**). Some mutations completely disrupted the secondary structure surrounding the epitope (cyan Asp-10 vs. pink Ala-10 in **d**). The predicted overall 3D structures of the synthetic E proteins were not dramatically different from their corresponding parental wild-type proteins.

As shown in Figure 2, the alanine substitutions that did not alter the overall predicted structure of the recombinant synthetic E proteins clustered in three separate and distinct motifs at the tips of Domain II (the fusion loop; Figure 2a,b), Domain III (Figure 2a,c) and I (Figure 2a,d). According to our molecular modeling analyses, alanine substitution altered the orientation of the lateral chains of the amino acid residues (Figure 2b–d), disrupted the secondary structure of the peptide chain (Figure 2b), or altered the orientation and extension of the antiparallel ß-sheets present in Domains I and III (Figure 2c,d). All the selected substitutions altered the solvent-accessible surface of the linear cross-reacting epitopes in the *in silico* 3D models.

### 3.2. Recognition of the Synthetic DENV E Proteins by a Monoclonal Anti-DENV-2 Antibody

**Figure 3:**
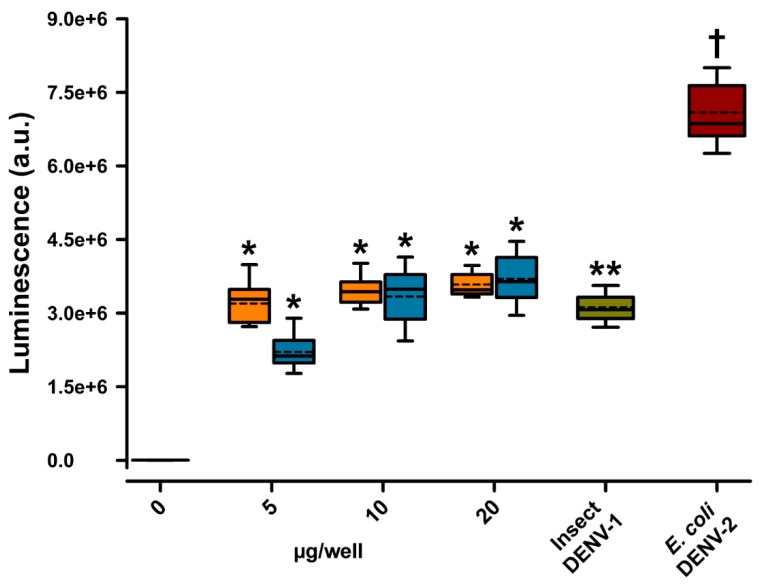
Recognition of synthDENVElope proteins by a monoclonal anti-DENV-2 antibody. A murine monoclonal (NYRDeng2) antibody directed against DENV-2 viral particles was capable of binding to both syDENV-1 (orange boxes) and -2 (blue boxes) proteins in an indirect ELISA test. Luminescence signals (arbitrary units) generated by this interaction were similar to levels generated when the antibody was incubated with the recombinant DENV-1 E protein produced by insect cells (dark green box) but were lower than signals elicited when the recombinant DENV-2 protein expressed in *E. coli* (dark red box) was present in the wells. Luminescence levels in the presence of any of the synthetic recombinant E proteins were at least 1000-fold higher (2 × 10^6^ vs. 0.8 × 10^3^ a.u.) than those obtained as base-line levels in the absence of any E protein in the wells (left-most box at 0 µg/well). * denotes *p* < 0.01 vs. 0 µg/well; ** denotes *p* < 0.01 vs. 0 µg/well and syDENV-2 5 µg/well; and † denotes *p* < 0.01 vs. all other groups.

Synthetic DNA sequences coding for these redesigned mutant E polypeptides were generated and two synthDENVElope proteins were expressed in *E. coli*. We then evaluated if they were capable of binding to a monoclonal antibody directed against DENV-2 viral particles (clone NYRDeng2) in a chemiluminescent ELISA test. As shown in Figure 3, this monoclonal antibody recognized the two synthDENVElope proteins, as well as the two recombinant wild-type DENV envelope proteins. Luminescence levels were similar to the ones generated when the antibody reacted with recombinant E protein from the DENV-1 produced in insect cells but were lower than those generated when reacted with recombinant E protein from the DENV-2 expressed in *E. coli*.

### 3.3. Sera from DENV NS1-Positive Patients are Separated into Distinct Groups through Their Interaction with SynthDENVElope Proteins

**Figure 4:**
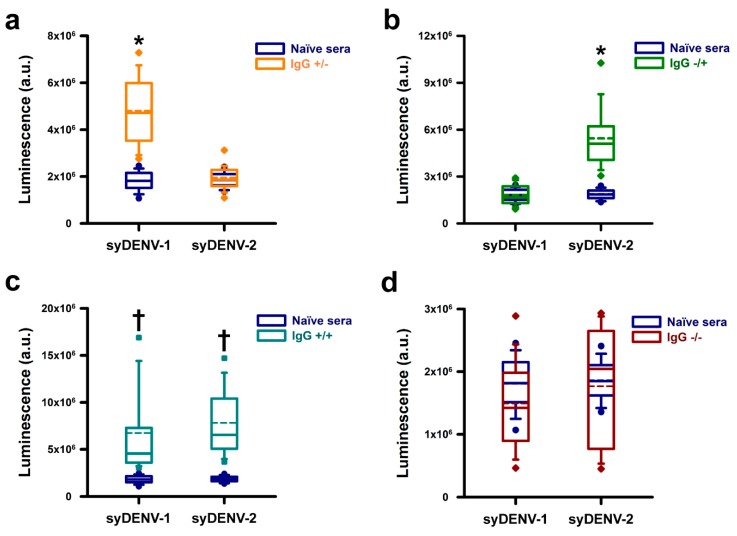
Immunoreactivity of DENV NS1+ positive sera against the synthDENVElope proteins. The IgG-antibodies present in NS1+ sera reacted against the syDENV proteins and their signals could be separated into distinct groups. The first, small group of NS1+ sera showed higher, statistically significant luminescence levels when reacting against the syDENV-1 protein than the syDENV-2 protein. These sera also showed higher, statistically significant signals than those generated by a panel of naïve sera reacting against both synthetic proteins (**a**). A second, larger, group of NS1+ sera generated higher, statistically significant luminescence signals when reacting against the syDENV-2 protein than the signals generated by naïve sera reacting against both proteins (**b**). The third, and largest, group of NS1+ sera showed statistically significant higher luminescence levels against both synthDENVElope proteins than the signals generated by the naïve sera (**c**). A small fraction of NS1+ sera generated luminescence signals that were not different from those generated by the naïve sera (**d**). * denotes *p* < 0.01 vs. all other groups; † denotes *p* < 0.01 vs. naïve sera.

Because we wanted to test the utility of our approach of synthetically generating DENV E proteins in diagnostic conditions, simulating the field conditions common to the Northeaster region Mexico, we evaluated a panel of 130 DENV NS1-positive sera in a chemiluminescent ELISA. We found that nearly 88% (114/130) of these sera contained IgG antibodies capable of binding to the synthDENVElope proteins. These DENV NS1-positive sera showed three different profiles of interaction with our mutant E polypeptides; specifically, a small fraction, roughly 9% (11/130), of them recognized only the syDENV-1 protein (Figure 4a), while a larger fraction, 36% (47/130), recognized only the syDENV-2 polypeptide (Figure 4b). 

The largest fraction, 43% (56/130) of the NS1+ positive sera contained IgG antibodies that generated signals of similar intensities when reacting against both syDENV-1 and -2 proteins (Figure 4c). These results correlate with the epidemiological data for the period when the sera samples were collected, because DENV-1 and DENV-2 were the only circulating DENV serotypes at the time. Only 12% (16/130) of the sera generated signals close to the base-line levels generated by a panel of 100 naïve sera (dark-blue boxes in all panels), as shown in Figure 4d.

### 3.4. Sera from PCR-Positive DENV-4 Infected Patients Did Not React with the SynthDENVElope Proteins

**Figure 5:**
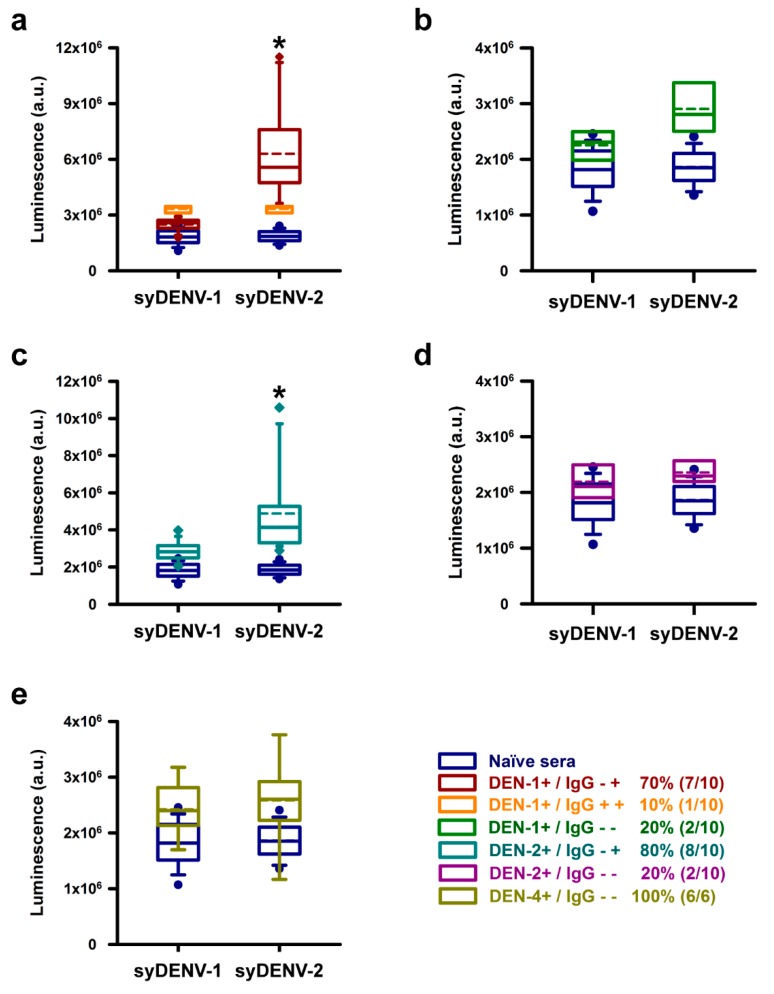
Reaction of sera from PCR-serotyped DENV-infected patients against the synthDENVElope proteins. Three panels of sera from NS1-IgG+ seroconverted patients that were previously serotyped by molecular methods were tested against the synthDENVElope proteins. Sera from confirmed DENV-1 infections were able to generate higher levels of luminescence against the syDENV-2 than against the syDENV-1 protein (dark red boxes in **a**), and one of the samples (orange boxes in **a**) reacted against both syDENV-1 and -2, generating higher luminescence signals than naïve sera in these tests (dark blue boxes in all panels). Two of these sera did not generate luminescence levels above those of the naïve sera (**b**). The sera from confirmed DENV-2 patients recognized only the syDENV-2 but not the syDENV-1 protein (cyan boxes in **c**). Two of these samples did not react against any of the syDENV proteins (magenta boxes in **d**). None of the sera from a panel of confirmed DENV-4 infections reacted against any of the synthetic DENV E proteins (golden boxes in **e**). * denotes *p* < 0.01 vs. all other groups.

We evaluated the serospecificity of the synthDENVElope proteins using three separate panels of seroconverted (NS1-IgG+) patients in which the serotype of infecting DENV was identified by molecular methods. Most of the sera (7/10) from patients infected by DENV-1 generated higher ELISA signals when reacting against the syDENV-2 protein than against the syDENV-1 protein and a small fraction (1/10) generated similar luminescence levels when reacting against both syDENV-1 and -2 proteins (Figure 5a). Twenty percent of these DENV-1 positive sera did not react with any of the synthDENVElope proteins (Figure 5b).

Most of the DENV-2 positive sera (8/10) recognized only the synthDENVElope-2 protein, and none of these sera generated high levels of luminescence when reacting against the synthDENVElope-1 protein (Figure 5c). As observed with the DENV-1 positive sera, 20% (2/10) of these sera did not recognize any of the synthetic mutant E proteins (Figure 5d).

Remarkably, none of the sera from the DENV-4-positive patients, obtained from Chiapas state in the southern region of Mexico, generated luminescent signals above the levels from naïve sera (Figure 5e).

## 4. Discussion

The serological diagnosis of flaviviral infections is limited by the cross-reactivity of the major antigenic determinants of this group of viruses. Most of the mosquito-airborne viruses, and their vectors, are endemic to regions of the world where economic development prevents the widespread use of molecular diagnostic methods that allow for the identification and serotypification of the infecting virus.

For DENV infections, knowing the previous history of infections in a patient is extremely important, because secondary infections by a different phenotype increase the risk of major hemorrhagic complications of Dengue [34,35]. Moreover, with the expansion of new flaviviral agents into areas that were not their usual niches, such as the introduction of the Zika virus into the Americas, the ability to distinguish between infections caused by the different flavivirus co-circulating in a population is imperative for health services around the world [1,2]. 

For these reasons, generating improved antigens that allow for the distinction between different flaviviruses and/or their serotypes by the use of serological methods would be extremely useful. Several strategies have been employed to modify the major antigenic determinants present on the surface DENV, mainly the M and E proteins. These strategies include the expression of truncated versions of the E protein, limiting the recombinant proteins to one of its three domains [36,37], and the inactivation of epitopes by amino acid residue substitution [26].

Here, we describe a strategy for designing synthetic sequence coding variants of the DENV envelope protein, where in silico substitutions of key amino acid residues constituting linear epitopes on the E protein are tested for their capability to alter the tridimensional conformation surrounding the linear epitope without disrupting the overall predicted structure of the recombinant protein. Some of the mutations correspond to previously described amino acid substitutions that inactivate cross-reacting epitopes such as those around the fusion loop on Domain II of the E protein, chiefly Trp-101 (Figure 1, middle panel; Figure 2b) [26], which is conserved by the members of the Flaviviridae family [31]. Inactivation of this fusion-loop epitope by alanine substitution leads to decreased immunoreactivity to monoclonal mouse antibodies directed against the flaviviral E proteins and would prevent cross-reaction between different members of this family [32].

This in silico modeling allows for the design of artificial DNA sequences coding for mutant E proteins and their expression in heterologous systems. By using recombinant proteins expressed in bacteria, we were able to evaluate the effect of inactivating several linear epitopes preserved by all DENV serotypes to cross-reactivity against sera from DENV-infected patients. We designed two synthetic proteins based on the sequences of the DENV E protein preserving the linear serotype-specific epitopes of DENV-1 (syDENV-1) or DENV-2 (syDENV-2) while inactivating most of the linear epitopes conserved along all serotypes of DENV [10,17,26,32].

Both synthetic DENV E proteins were recognized by a commercially available monoclonal antibody directed against whole DENV-2 virions (clone NYRDeng2) (Figure 3), generating lower luminescence levels in an indirect ELISA test than the wild-type recombinant DENV-2 E protein expressed in bacteria but similar luminescence levels to the wild-type recombinant DENV-1 E protein expressed in insect cells (Figure 2 rightmost boxes). Because the epitope recognized by this monoclonal antibody has not been characterized, it is not clear if it binds to a linear or structural epitope, but this result suggests that the overall conformation and immunoreactivity of our synthetic proteins is not much different from that of other available recombinant DENV E proteins and that the substitutions we introduced can decrease binding to an antibody directed against a specific DENV serotype.

The mutant synthetic DENV E proteins were also able to bind to the IgG antibodies present in the sera of DENV NS1+ patients because nearly 88% (114/130) of these serum samples generated ELISA signals higher than those of a panel of naïve sera (Figure 3). Most of these sera (103/130, roughly 79%) were able to recognize the mutant synthDENVElope-2 protein, which correlates with a higher incidence of DENV-2 infections in Nuevo Leon state in Mexico during the period when samples were collected (https://www.gob.mx/salud/documentos/direccion-general-de-epidemiologia-panorama-epidemiologico-de-dengue-2015-semana-epidemiologica-52). 

Nearly half (56/103) of the sera samples that bound to the syDENV-2 protein also bound to the syDENV-1 protein. These results, taken together with the results obtained in the ELISA experiments using a monoclonal DENV-2 antibody (Figure 2), indicate that some of the cross-reacting epitopes remain intact in our syDENV-2 protein and/or that the overall structure of this protein is better preserved than that of the syDENV-1 protein. Our experiments with the sera obtained from PCR-serotyped, seroconverted patients also suggest that the antigenicity of syDENV-2 is better preserved because nearly all DENV-1 and -2 positive sera were able to generate higher levels of luminescence in our ELISA test when reacting against the syDENV-2 protein than when reacting against the syDENV-1 protein (Figure 4a–d). Whether the remaining cross-reacting epitopes present in the syDENV-2 protein represent linear or structural epitopes remains to be determined. 

However, we were able to identify the NS1+ sera that selectively recognized just one of the syDENV proteins, either syDENV-1, which represents the smallest fraction (11/130 sera) of syDENV-positive sera in our sample, or syDENV-2, which represent a larger fraction (47/103) of the NS1-+ sera. These results suggest that it is possible to improve the serospecificity of recombinant DENV E proteins by using synthetic biology strategies. This scenario is also supported by our results obtained by analyzing the interaction of sera from PCR-positive DENV-4 seroconverted because these sera samples were not able to bind to any of our synthetic DENVElope proteins.

Our results show that substituting key residues for alanine within linear epitopes on the surface of the DENV E protein abolishes the contribution of some cross-reacting epitopes to its antigenicity, improving is utility for serological diagnostic methods. The modification of structural epitopes could further improve the contribution of serotype-specific epitopes, which could lead to the development of better antigens for serological determination of infecting DENV serotypes and/or discrimination among different flavivirus, such as the Zika virus.

The use of this kind of strategy would allow for the generation of serospecific synthetic antigens that can be expressed in bacterial systems. The use of the improved DENV antigens in serological diagnostic methods would decrease the complexity and costs incurred by the current PCR-based gold standard methods of DENV serotyping, which are not always available in rural areas of the world.
